# Balancing scientific interests and the rights of participants in designing a recall by genotype study

**DOI:** 10.1038/s41431-021-00860-7

**Published:** 2021-05-13

**Authors:** Deborah Mascalzoni, Roberta Biasiotto, Max Borsche, Norbert Brüggemann, Alessandro De Grandi, Martin Goegele, Sara Frygner-Holm, Christine Klein, Maria Kösters, Ciara Staunton, Peter P. Pramstaller, Michael Krawczak, Andrew A. Hicks

**Affiliations:** 1 Institute for Biomedicine, Eurac Research, Affiliated Institute of the University of Lübeck, Bolzano, Italy; 2 Department of Public Health and Caring Sciences, Center for Research Ethics and Bioethics, Uppsala University, Uppsala, Sweden; 3 Institute of Neurogenetics, University of Lübeck, Lübeck, Germany; 4 Department of Neurology, University Medical Center Schleswig-Holstein, Lübeck, Germany; 5 Department of Neuroscience, Uppsala University, Uppsala, Sweden; 6 School of Law, Middlesex University, London, UK; 7 Institute of Medical Informatics and Statistics, Kiel University, Kiel, Germany

**Keywords:** Ethics, Genetics research

## Abstract

Recall by genotype (RbG) studies aim to better understand the phenotypes that correspond to genetic variants of interest, by recruiting carriers of such variants for further phenotyping. RbG approaches pose major ethical and legal challenges related to the disclosure of possibly unwanted genetic information. The Cooperative Health Research in South Tyrol (CHRIS) study is a longitudinal cohort study based in South Tyrol, Italy. Demand has grown for CHRIS study participants to be enrolled in RbG studies, thus making the design of a suitable ethical framework a pressing need. We here report upon the design of a pilot RbG study conducted with CHRIS study participants. By reviewing the literature and by consulting relevant stakeholders (CHRIS participants, clinical geneticists, ethics board, GPs), we identified key ethical issues in RbG approaches (e.g. complexity of the context, communication of genetic results, measures to further protect participants). The design of the pilot was based on a feasibility assessment, the selection of a suitable test case within the ProtectMove Research Unit on reduced penetrance of hereditary movement disorders, and the development of appropriate recruitment and communication strategies. An empirical study was embedded in the pilot study with the aim of understanding participants’ views on RbG. Our experience with the pilot study in CHRIS allowed us to contribute to the development of best practices and policies for RbG studies by drawing recommendations: addressing the possibility of RbG in the original consent, implementing tailored communication strategies, engaging stakeholders, designing embedded empirical studies, and sharing research experiences and methodology.

## Introduction

The unprecedented amount of human genomic data generated by next-generation sequencing (NGS) in different research settings, including population biobank projects, is an important resource to advance our understanding of both health and disease. However, the richness of the genetic data now attainable through NGS calls for similar levels of detail in terms of the accompanying phenotype(s). In this context, re-inviting or recruiting research participants for deeper phenotyping, based upon the presence or absence of certain genetic variants in an approach termed “recall by genotype” (RbG) or “genotype-driven research recruitment”, is gaining increasing importance. The rationale for RbG is that drawing upon the potential biological impact of the variants in question already at recruitment can render the subsequent phenotyping process more specific and cost-effective [1].

The process of re-inviting individuals who already participated in a research project may seem unproblematic at first glance, but it nevertheless poses a challenge to established ethical, legal, and practical research frameworks, and therefore warrants further consideration [2, 3]. In fact, patient-based medical research generally proceeds under the proviso that a potential study participant receives all the information necessary to make an autonomous decision about their participation, or not, during the enrolment phase of the study. This also holds legal constrains in Italy where genetic data can only be communicated based on the individual informed choice and often genetic counselling is required [4]. When participants are approached because of their genotype, however, communicating the reason for the contact may already entail the disclosure of sensitive information that the individual may not actually wish to know [3, 5–7].

Designing an RbG process requires several decisions to be made upfront [2]. First, the genetic details to be communicated to the study participants must be determined. Whether a particular genotype is problematic to report depends upon whether such genotype is known to have a deleterious phenotypic effect or not or whether it comprises a new, hitherto unknown, variant. Second, the means must be provided to ascertain the participants’ preferences about the feedback of potentially problematic information. Both these issues relate to the ongoing debate about the extent to which researchers are obliged to return individual research results to study participants. In genetic research, study subjects are usually informed beforehand about the type of variants to be analysed and, in the course of consenting, can be given some form of counselling allowing them to weigh up the pros and cons of receiving individual research results [8–11]. In the context of RbG, careful consideration must therefore be given to the informed consent process, particularly to the kind of information provided about future research and whether it potentially involves RbG [6, 12, 13].

The number of studies relying upon RbG can be expected to increase considerably in the near future, and several ad hoc cohorts (e.g. the Oxford biobank [14]) have been set up specifically for pursuing this type of research [1]. Members of such cohorts should be aware of the possibility that they may be re-contacted for further research, based upon their genetic make-up. On the other hand, in population studies where RbG was not foreseen at the outset, re-call could be performed without revealing the main reason for it. While such an approach may appear unacceptable on principle, currently, there are no recognized ethical and legal standards for RbG in cohorts where RbG was not planned and which was therefore not included in the original informed consent [2, 6, 15, 16].

The Cooperative Health Research in South Tyrol (CHRIS) study is a longitudinal cohort study targeting the general population of Val Venosta in South Tyrol, Italy. CHRIS was launched in 2011 to investigate the molecular basis of common chronic diseases associated with human ageing and to analyse the interaction of genetic factors with life-style and environmental factors [17]. Baseline assessment in CHRIS comprised screening of the cardiovascular, metabolic, neurological, and psychosocial health status of 13,389 participants, representing 45% of the adult inhabitants of the valley. The biobank of the CHRIS study includes a vast collection of biosamples (blood, urine, cells, DNA). Genetic data obtained from the DNA can potentially be used in several studies, including also RbG studies, which were not planned at the time of recruitment. CHRIS promotes a participant-centric approach by considering the interests, needs, and demands of participants at all the steps of the research study [17, 18]. With the growing demand for RbG studies, we realized that CHRIS needed to develop an ethically acceptable and legally sound RbG framework. The aim of the present paper is to describe the steps taken to develop such a framework, by designing and implementing a pilot RbG study and by showing how we addressed the ethical issues and the legal requirements associated with RbG in the process. Moreover, our experience with an RbG pilot study in CHRIS allowed us to draw some general recommendations for policy development in such studies.

## Workflow

When designing the pilot RbG study, we decided to also embed within it an empirical study, including qualitative and quantitative parts. The empirical study was designed to collect direct feedback from participants who were involved in an RbG study. This was important not only for understanding participants’ experience while participating in our pilot RbG study (feedback on the recruitment and communication strategy) but also for understanding their views on RbG-designed studies in general (interest in participating, preference regarding re-contact, and concerns). The pilot RbG study and the embedded empirical study were conducted in August 2018 at the CHRIS centre in Silandro/Schlanders, Italy. The studies are a joint effort by Eurac Research, Institute for Biomedicine, Bolzano, Italy, and the University of Lübeck, Germany, made as part of a wider scientific project on reduced penetrance in movement disorders (ProtectMove). The pilot study comprised a clinical examination (neurological function assessment), whereas the empirical study consisted of two questionnaires (one administered before the clinical examination and the other after the clinical examination and before leaving the study location) and an interview (conducted after the clinical examination). The whole study (comprising the pilot and the embedded empirical study) was performed in accordance with the Declaration of Helsinki and was approved by the ethics committee of the Azienda Sanitaria dell’Alto Adige. All participants provided written consent to participate in the study. Here, we describe the design of the pilot study and the policy building process, drawing on some preliminary observations made in the empirical study which shed light on the RbG process. The methodological details and the results of the empirical study will be published elsewhere.

**Fig. 1:**
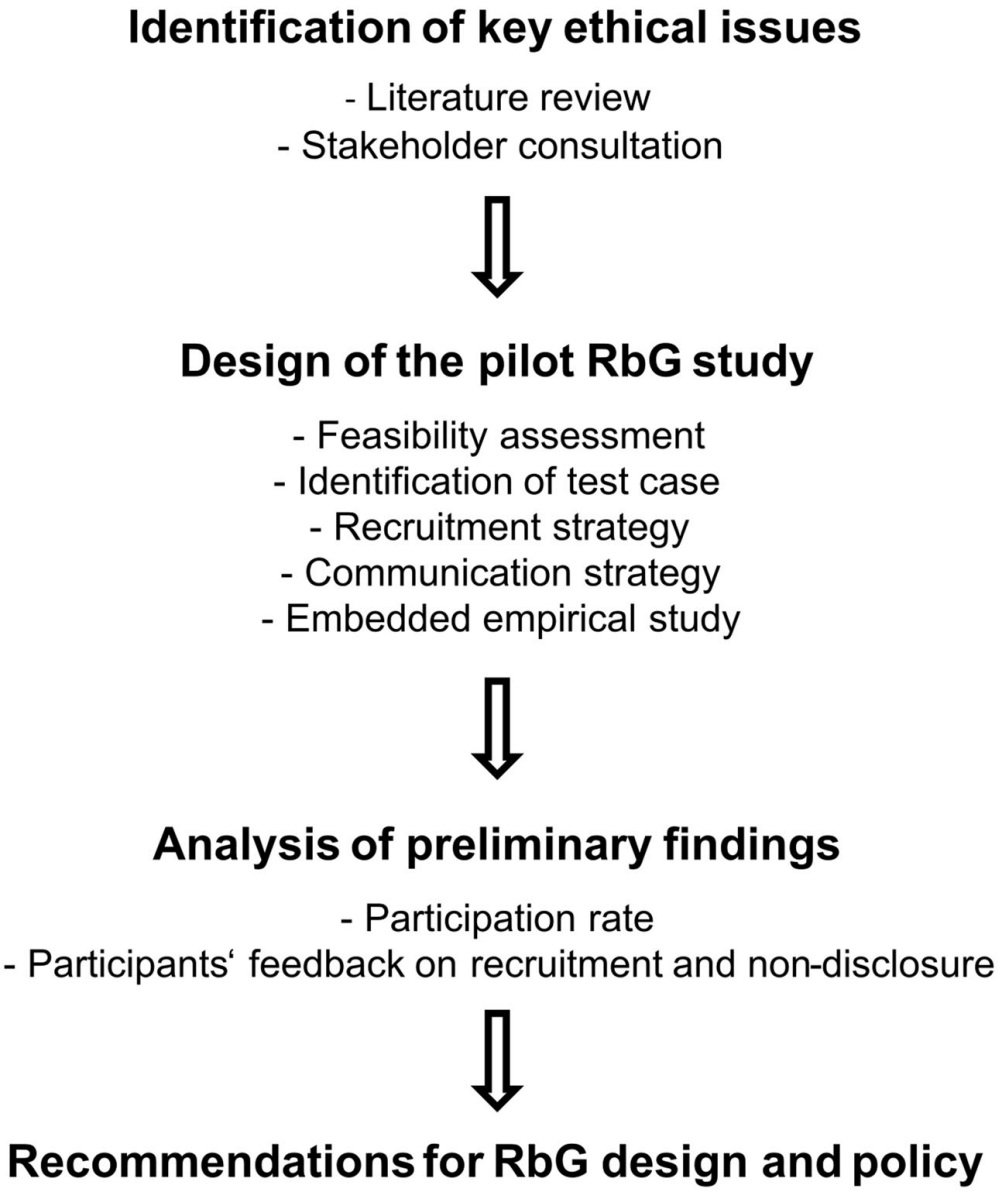
Design of the pilot RbG study and RbG policy building process in CHRIS. The figure summarizes the workflow of the present study, showing a multi-step process as a model for RbG study design and policy development.

The stepwise strategy we took in order to conduct a pilot RbG study in the frame of the CHRIS study is shown in Fig. 1. Through a narrative literature review, we identified the key issues associated with RbG studies, and the practical recommendations that were drawn from on how to conduct an RbG study in an ethically acceptable way. In the design of both the pilot study and the embedded empirical study, we took into account the views and suggestions of different stakeholders (clinical geneticists, general practitioners (GPs), ethics committee, CHRIS participants). In details, we discussed with clinical geneticists in order to identify legally sound and ethically sustainable strategies for the communication of genetic information; we consulted the ethics committee and GPs to ensure we included measures to safeguard participants in the pilot study; the experience of an earlier empirical study on return of results conducted with CHRIS participants (own unpublished observations) allowed us to design the questionnaire for the embedded quantitative study. The design of the pilot included a feasibility assessment, the identification of the test case, the choice of the recruitment strategy, and a communication strategy.

## Identification of relevant RbG ethical issues

In the narrative literature review, papers focused on the ethical aspects related to RbG (from here referred as “ethical RbG papers”) and papers focused on the biomedical and clinical aspects of RbG-designed studies, aiming at understanding the phenotypic effect of specific genetic variants (from here referred as “biomedical RbG papers”) were reviewed. We sought to understand how to balance scientific interests and participants’ rights. We expected to find, in the biomedical RbG papers, the translation into practice of the ethical issues discussed in the ethical papers, and approaches to the challenges that an RbG-designed study poses.

The ethical papers were mostly focused on the perspective of participants to RbG: authors investigated participants’ understanding of the rationale of RbG-designed studies [15], the experience of participation in genetic research [15, 19, 20], the expectations associated with and the experience of recruitment in RbG studies [19, 21], and the communication of individual genetic results (meanings of genetic results, disclosure) [15, 20–23] (Table 1).

**Table 1:** Participant perspective on RbG issues according to the reviewed literature.

	Findings	Recommendations for researchers
Understanding the rationale of RbG-designed studies	–Participants had basic knowledge of genetics and limited interest in specific study designs (e.g. RbG) [15]. –Former participants in an RbG study did not realize that their recruitment was based on being carriers of genetic variants of interest for researchers found through a previous study, and did not recall that they received their individual research results [19, 22].	–Researchers should highlight the complexity of scientific research by offering participants suitable information and opportunities for reflection [15]. –In order to avoid misunderstandings, researchers should explain to participants notions of research study design [19]. –During recruitment in paediatric research, researchers should address potential benefits and risks for all the possible stakeholders [20]. –Researchers should clearly communicate to participants the current state of knowledge in their field of research [21].
Experience of participation in genetic research	–A general positive view on research participation overall by study participants was based on trust, solidarity, and reciprocity, which developed through the long-term relationship with the study and participant’s expertise as data provider [15]. –Patient-participants and general population biobank participants understood their roles as research participants differently [19]. –Possible benefits for their child and for other stakeholders, altruism and positive action were the main motivations for the participation in genetic research of parents of children with a disease [20]. –Parental perception of the participation-associated risks and benefits for their child differed according to the type of disease with which their children were affected [20].	–Researchers should consider the burden (e.g. time needed, inconvenience) on the parents/family of minors with a disease, when the minor participates in research [20].
Expectations associated with and the experience of recruitment in RbG studies	–Patient-participants and general population biobank participants perceived their recruitment in RbG studies differently [19]. –Based on altruism and a positive attitude towards research, participants found re-contact for research recruitment and for RbG-designed studies acceptable [21]. –Participant preference for being re-contacted for future research varied according to study population and study design [21]. –Trust played a role in the acceptability of re-contact [21].	–The possibility of re-contact for further research recruitment should be disclosed in the original informed consent [20]. –When designing RbG studies, researchers should know their study population well in order to develop tailored research practices [21]. –Researchers should plan recruitment according to context (general population or disease-specific cohort; conditions in the original consent; access to samples and data) [22]. –Contextual factors and empirical evidence should inform the design of genotype-driven recruitment [21]. –When designing RbG studies, researchers should consider prospective participants’ views, assumptions, and expectations [21].
Communication of individual genetic research results	–Participants’ status (e.g. belonging to a disease-specific cohort or to a general population biobank) influenced the perceived meaningfulness of genetic research results (validity and utility), views about the return of individual genetic research results, and desire of receiving individual research results [22]. –Patient-participants expressed layered expectations about return of results: individual genetic research results were assumed to give answers to questions about specific issues (reasons for the researchers interest in a specific participant, knowledge of the disease under study or the participant themselves, heritability of the disease, possible actions) [23]. –Patient-participants’ perception of risks and benefits of disclosure of individual genetic research results may be different compared to that of population study participants [21]. –Parents of children with a disease were interested in receiving individual genetic research results about their children for clinical utility, reproductive planning, explanation of the etiology of the disease, proactive behaviour change [20]. –The return of individual genetic research results might have a psychological impact on parents of children with a disease [20]. –Parents of children with a disease would like to be able to decide about receiving or not their child’s individual genetic research results [20]. –Parental perception of the risks and benefits of receiving individual genetic research results of their children differed according to the type of disease with which their children were affected [20]. –Participants’ views on disclosure of individual genetic research results varied according to study type [21]. –Participants showed interest in receiving information on aggregate research results to be updated about the study and for reciprocity reasons [21]. –Participants showed moderate interest in receiving communication of research outcomes [15]. –Participants’ low expectations of receiving individual research results relied on the acceptance of the non-disclosure policy of the study [15].	–The recommended guidelines for returning only results of clinical utility may not be the most suitable approach in RbG studies [21]. –The nature of the results (validity and utility) and the context (participant population and participant’s relationship with researchers) should be considered in the decision of whether or not to disclose the individual genetic research results [22]. –If individual or aggregate genetic research results are disclosed during recruitment, researchers should thoroughly explain to patient-participants the nature of their research findings and any limitations [23]. –In the case of disclosure of individual genetic research results during the recruitment process, researchers should provide clear and appropriate written and oral explanations, by taking into account possible meanings associated with those results [22]. –In case individual genetic research results are offered to parents of children with a disease, researchers should clearly explain the meaning and validity of such results [20]. –Researchers should investigate (e.g. through participants’ focus groups or by addressing physicians or patient advocacy organizations) and identify the expectations of patient-participants associated with individual or aggregate genetic research results [23]. –Any decision about the communication of individual research results should be taken by considering participants’ needs and perspectives, which can be acknowledged through participant engagement in governance or involvement in empirical studies [15].

Importantly, they highlighted that the “context” may influence the steps to be taken while designing RbG studies, especially in the process of re-contact and the communication (or not) of genetic research results. The discussed contextual factors included:

1. The participant’s relationship with the researchers involved [22, 24].
2. The modality of re-contact, i.e. in which way and by whom the re-contact is made [24].
3. The nature of the target participants population (general population or disease-specific cohort) [21–23].
4. The possible involvement of other family members [24].
5. The focus of the RbG study compared to that of the original study [24].
6. For participants from a disease-specific cohort or for parents of minors with a disease: disease status, type and nature of the condition being studied, current knowledge about the condition, possible expectations and meanings associated with research results [23], time, or convenience factors involved in participating [20].

The perspective of other stakeholders, like institutional review board chairs, has been addressed as well by Beskow et al. [24]. The authors found that “there is unlikely to be a “one-size-fits-all” solution, but rather several ethically acceptable approaches to genotype-driven recruitment depending on context” [24]. This paper highlighted the importance of addressing the re-contact for further research in the original informed consent (also suggested by Beskow et al. [21]), of using a lay language and clear communication with participants about current knowledge in the research field that they are involved in (also suggested by Minion et al., and Michie et al., and Beskow et al. [15, 19, 21] respectively), and of the clinical validity of research results to be communicated [24]. As a whole, these studies showed the unresolved tensions in RbG studies and provided both solutions and recommendations (see Table 1, and recommendations suggested by Beskow et al. [2]). The design of a generalized RbG policy is difficult due to the complexity of the context, as described above.

RbG studies are often conducted as sub-studies within the framework of a larger-scale research study, which can be longitudinal, long term, biobank based, and with broad aims. The most recently published RbG studies included in our literature review were conducted in Europe, mostly in the frame of population studies such as the Cambridge BioResource [25, 26], the Avon Longitudinal Study of Parents and Children [27, 28], the Exeter 10000 study [29], the Estonian Biobank of the Estonian Genome Center [30], the PPP-Botnia study [31], and other types of study [32, 33]. These RbG studies were designed to understand a variety of diseases, conditions, and topics, such as inflammatory bowel disease [25], arterial and venous diseases [26], schizophrenia [27], cardiovascular disease [28], level of adiponectin [29], familial hypercholesterolaemia [30], type 2 diabetes [31], anxiety disorders [32], and predisposition to hypertriacylglycerolaemia [33]. Where the variant(s) under study had clinical implications and treatment options for the participant and their relatives, genetic counselling, the disclosure of the carrier status, and further tests for family members were offered [30]. We observed that, in general, details on the type of informed consent and re-contacting strategy were usually only briefly described. On the other hand, when discussing the choice of RbG design, authors of biomedical RbG papers largely justified their approach with scientific arguments (e.g. statistical efficiency and power, ease of data collection, and maximizing resources [32–37]), thus demonstrating more of a focus on the researchers perspective. Based on this observation (the perceived lack of discussion of the ethical issues at stake when conducting RbG studies), we decided to embed an empirical study into the pilot RbG study, in order to directly collect feedback on the research study from the participants, with a view to improving our study design and be better placed to address participants’ views in the future.

In both RbG studies and the return of research results to study participants, the disclosure of potentially unwanted or distressing information during re-contact might occur, necessitating a reflection on how best to handle the communication with participants In view of this commonality, we drew upon the findings obtained from a previously conducted qualitative study with CHRIS participants on the return of results (own unpublished observation). In order to tackle the issue of disclosure in a nuanced way and to meet the expectations of participants, data from the return of results interviews were used as a basis to develop the questionnaire for the empirical study embedded in the pilot RbG study.

To enrich the key ethical issues identified in the literature with the clinical experience of experts, we engaged in a discussion on RbG with nine clinical geneticists, working at the hospitals in Bolzano, Verona, and Bologna. We sought their view because it is of relevance for the medical and ethical implications of reporting genetic information to study participants. Specifically, D.M. and M.K. discussed possible strategies for communicating genetic findings with members of the Clinical Genetics Unit in Bolzano, which will play a vital role in future RbG studies in CHRIS because it is assigned the task of providing necessary genetic counselling within an RbG framework, under specific conditions. Together with the Bolzano Clinical Genetics Unit, we identified four scenarios of inherited genetic disease (for breast cancer, Parkinson’s disease (PD), Huntington’s disease, and malignant hyperthermia), to serve as a simplified representation of the complexities of interpretation for a genotyping result in terms of associated pathology. The four diseases differed by mode of inheritance, genotype-associated disease risk, age at onset, prevention, treatment, and clinical severity, thereby providing an acceptable typology model of genotyping results. This model has been used in a return of research results study to facilitate the discussion on the views on the return of individual genetic results, by showing a panel of results which will have a very different impact on health: for example, participants were asked whether they would like to receive their genetic results about diseases such as Huntington’s disease, where being carrier of the responsible gene variant results in 100% risk of developing the disease, 50% risk of transmitting the disease to their children, and for which there is no cure and no prevention. Each disease served to represent a different scenario (own unpublished observations). The model was occasionally used during the interviews conducted in the empirical study reported here, if the conversation raised the opportunity. In this case, it served as a summary of the variables that participants might find relevant in their decision in participating in research about genetic diseases, and to stimulate reflection on the impact that the information received during recruitment and participation may have (e.g. emotional impact). The description of the diseases in this model has been integrated into the information provided for the CHRIS study informed consent, revised for the first follow-up phase (commenced October 2019) [38].

Through the steps described above, we identified several key points to be addressed both in the design of our pilot RbG study and also in a potential general RbG policy:

1. The option for re-contact for further research inserted into the informed consent (to legally be able to re-contact participants according to their autonomous choice).
2. To address the return of individual research results in the informed consent where individuals make an autonomous specific choice addressing the willingness to receive information on their genetic data, explicitly addressing the right to know and not to know.
3. The availability of information about the genetic basis of re-contact for RbG studies that addresses the right to be informed and provides the basis for an informed choice.
4. A clear communication on the rationale for returning (or not) research results for transparency, and again to ensure that individuals are aware of the process.

## Design of the pilot RbG study

### Feasibility assessment

For an RbG study to be acceptable in the first place, any re-contact must be covered by the consent given at the time of recruitment. In the original CHRIS informed consent form, which is a dynamic-based consent [17], participants could choose among different options for re-contact. There are two points in this consent that addressed the re-contact, each with a different purpose. In one option, participants could decide whether they wanted (or not) to be re-contacted for communication and/or further studies. In the other option, they could decide if they wished to be informed about research results, including genetic results. In the latter case, they could indicate their preferences through a series of options: they could choose either to be informed (right to know) or not to be informed (right not to know) or to be informed only if actionability is possible (screening, therapy, implications for family planning) or to be informed only if results potentially affect their family’s health.

We concluded that legally and ethically the concomitant choice of the options “agree to re-contact for communication and further studies” and “agree to re-contact for incidental genetic findings” is in fact tantamount to approving re-contact by genotype and, hence, meets eligibility requirements for invitation to an RbG study.

Through a discussion with the ethics committee of the Azienda Sanitaria dell’ Alto Adige and with local GPs, we agreed on the feasibility and upon additional measures to safeguard participants in the pilot study. First, the pilot study should be focused on genetic variants with moderate to low disease penetrance. Second, participants should be provided further clarifications, if they so wished, in a timely and easily accessible way.

### Test case: heterozygosity for Parkin gene variants

Recent major sequencing efforts have revealed a surprisingly large number of carriers of genetic variants reported to cause disease under different modes of inheritance, who do not exhibit overt symptoms of disease [39]. With individuals carrying many dozens of such function-altering variants, an accurate estimate of genetic penetrance is important to measure. For movement disorders, one of the aims of the German DFG funded ProtectMove Research Unit is to examine reduced penetrance for Parkin gene (*PRKN*) variants usually disease-causing when inherited recessively. Such biallelic disease-causing *PRKN* variants (in a homozygous or compound heterozygous state) are causative of PD through a recessive inheritance pattern, with highly variable age of onset. Moreover, recessively inherited heterozygous disease-causing variants in genes for one disease may present as risk factors for PD. Such is the case for biallelic variants in the glucocerebrosidase gene (*GBA*) which cause Gaucher disease, whereas heterozygosity at the same position strongly predisposes to PD. This and other examples provide support for the notion that disease-causing variants with reduced penetrance and genetic risk factors are sometimes different expressions on a continuum of effect sizes rather than representing a clearly dichotomous situation. Notably, penetrance of *GBA* variants was estimated as 7.6, 13.7, 21.4, and 29.7% at 50, 60, 70, and 80 years, respectively [40]. Biallelic *PRKN* pathogenic variants cause recessively inherited PD with high penetrance and typically earlier onset, whereas heterozygosity only leads to a modestly increased risk for PD [41, 42]. Given that heterozygous *PRKN* variant carriers can develop disease, the age of onset of carriers of heterozygous *PRKN* variants has been shown to fall in between that of biallelic variant carriers (earlier AAO) and sporadic PD patients (later AAO), and imaging and neurophysiological studies have revealed alterations in unaffected carriers of heterozygous variants (for review, please see [41]), these observations provide the rationale to explore penetrance of heterozygous pathogenic variants in *PRKN*, especially considering their frequency in the population, which for variants predicted to be pathogenic when inherited recessively, is ~3% in CHRIS (own unpublished data), which is much higher than the prevalence of PD [43].

Following the incorporation of additional measures established with the ethics board and local GPs for participant safety as detailed earlier, heterozygosity for *PRKN* variants that can cause disease when inherited recessively, or potentially dominantly with very low penetrance, was chosen as a test case for the pilot RbG study.

### Recruitment

Our pilot RbG study followed a matched recruitment design with 25 pairs of *PRKN* variant carriers and matched non-carriers. Each pair was chosen to be closely related, of the same sex, and of similar age where possible. This approach was taken for two main reasons, satisfying both the measures to safeguards participants and the clinical aim of the pilot. Inviting carriers and non-carriers at the same time without telling them their individual status potentially reduces psychological strain. A matched recruitment design reduces the risk of ascertainment bias due to possible pre- or sub-clinical effects of *PRKN* gene variants.

### Communication strategy

German was used for study communication, as it is the preferred language among CHRIS participants [17]. Invitees were sent an invitation letter and an information brochure (for the original documents, see Supplementary Information 1). In the information material, we explained the study and its implications in lay language, aiming for clarity and easy understanding of the contents by the reader. For example, we avoided the use of gene symbols such as “*PRKN*” and preferred “Parkin gene” instead. For the same reason we avoided scientific jargon, e.g. we used “standard” instead of “wild-type” when describing the gene. The brochure was structured in the same way as the letter but included more details relative to each section. In the letter, we reminded the invitees of their participation in the CHRIS study and of their expressed interest in participating in further research (by previously agreeing to that in the CHRIS informed consent). Both the letter and the brochure illustrated the following points:

1. The aim of the study, namely to carry out a deep neurological examination to investigate protective mechanisms against neurological diseases, with a focus on PD.
2. The concept of reduced penetrance and the need for investigating the phenotype associated with a particular *PRKN* genotype.
3. The study design, i.e. two groups of participants were invited, with members of one group carrying the wild-type *PRKN* gene and members of the other group carrying variants of the gene.
4. That only results relevant for the participant’s health would be communicated. Their *PRKN* carrier status would not be disclosed as no clinical benefits nor direct individual consequences are currently known.

We concluded the letter by announcing an upcoming phone call from the CHRIS study assistants to fix an appointment and the contact details (phone number) of the CHRIS study for further questions and clarifications.

Since the consequences of *PRKN* gene heterozygosity are mostly unknown, we disclosed only the type of genetic variant under study, but not the carrier status of the addressee. We decided that the communication of the individual carrier status, with the uncertainty regarding clinical implications, would potentially cause distress to participants. In the information material, we clarified that current findings suggest that *PRKN* heterozygosity may increase the risk of neurological symptoms. We highlighted the possibility that the addressee could be carrier of either status. To comply with the additional measures to safeguard participants described above, a direct telephone number for answering further questions and for formal enrolment was made available from the date the invitation letters were sent out. Consent was obtained at the study centre, where explanatory material was also available alongside the information provided verbally by study nurses and doctors. If participants wished to ask questions or requested further clarification during the medical examination, a specialist medical doctor was available to respond. If specific outcomes of the clinical examination required medical follow-up, the participant was referred to suitable specialists.

## Preliminary data and findings

### Participation

Out of the 58 CHRIS participants invited by mail to participate in our RbG study, 50 (86%) agreed. The main reasons for non-participation were availability during the week of the pilot study. An additional relative of one carrier, interested in undergoing the clinical examination, asked also to be included, to which we agreed, but did not include their data in the subsequent analysis because of the self-referral.

### Feedback from participants

As a preliminary finding within the empirical study, most participants reported to be at ease with the recruitment process and with the non-disclosure of their carrier status. They were satisfied that they had the chance to have their concerns duly addressed. Only one person experienced anxiety when receiving the invitation to participate, but was reassured in a phone conversation with a study assistant.

## Lessons learned and recommendations for RbG policy development

### Context

The literature review revealed that when designing an RbG study, it is key to consider the context of the study. The dynamic consent model adopted by CHRIS facilitates the ongoing communication between researchers and participants, and offers an easy way of informing CHRIS participants about new study approaches, such as RbG. In our experience, CHRIS participants demonstrate a general positive attitude towards research and participation and trust towards the study (own unpublished results). The focus of the pilot RbG study (research into the mechanisms of PD) fits well into the main scope of the CHRIS study, i.e. the study of cardiovascular, neurological, psychiatric, oncologic, and metabolic conditions. These aspects combined might have played a role on the very high participation rate in the pilot and on the generally positive attitude towards participation. The participation rate of the RbG pilot study was slightly higher than that of other sub-studies conducted in the frame of the CHRIS study (own unpublished observation).

### Feasibility, re-contact, and communication

The framework of re-contact very much depends on whether re-contact was envisaged during the consent process of the original study. Based on the experience from our pilot study, we think that the possibility of future RbG studies should be addressed in the consent of original cohort studies as standard because RbG studies might entail disclosure of sensitive data and individuals have the right to refuse this information or to express the willingness to be informed. Along with appropriate information, this would provide cohort members an introduction to the concept, and an early chance to decide whether or not they want to be involved in such studies in general. At the time of the pilot study, the informed consent of the CHRIS study addressed the return of research results with several options, and did not specifically refer to RbG. We concluded, however, that the concomitant choice of accepting to be re-contacted for communication and further research and agreeing to be re-contacted with research results (relevant for health) by the participant was acceptable as a condition for invitation to an RbG study also in terms of legal requirements. When the first follow-up phase of the CHRIS study started in 2019, the information material of the informed consent was enriched with specific examples for the typology of genetic diseases that may form the basis of such further RbG studies, developed together with genetic counsellors, in order to offer participants a more nuanced perspective on the meaning and implications of carrying particular genetic variants.

The ideal framework of the information process, in a general consent setting, should aim to balance between the practicability of the consent procedure and the amount of provided information. We recommend that the communication strategy be adjusted to the significance of the variant(s) in question, e.g. while the carrier status for a variant of unknown significance may be communicated by mail, professional genetic counselling is required for variants with severe pathogenic implications. In Italy, the communication of individual genetic results, especially in the case of disease-causing implications, is usually done through genetic counselling. In this context, the role of informed consent is to pre-assess the willingness of people to be involved in an informational procedure by genetic counsellors, in case variants are found for RbG. This means that, in some cases, individuals should be contacted for proper disclosure/non-disclosure by genetic counsellors, according to clinical severity and actionability. Considering that the *PRKN* variants under study in our pilot RbG study are usually highly penetrant in a recessive inheritance mode and that we were studying heterozygous carriers with much reduced, or negligible penetrance, we did not set up any professional genetic counselling, which would be required as part of a study for variants with dominant inheritance and severe pathogenic implications. All participants in our pilot reported to be at ease with the recruitment strategy used. We also recommend including, as part of the setup of an RbG study, a point of direct contact (preferably by telephone) for potential participants, in order to collect and respond to potential queries and worries immediately. One invitee in our pilot study made use of this opportunity after receiving the invitation, and their concerns were sufficiently addressed.

As yet, no clear guidelines exist regarding the type of genetic variants that may be reasonably disclosed to participants in RbG studies. In our pilot study, we disclosed the genetic variant under investigation to invitees, but not their individual carrier status. This approach was met with ease by the participants. It may be possible that with the specific test case chosen for our pilot study (i.e. heterozygosity for low-penetrance *PRKN* variants), variant-based re-contact generates less anxiety than when being confronted with information of variants with a demonstrated major impact on health. The expected low clinical impact of these variants is also a likely explanation of why most participants deemed the non-disclosure of their carrier status acceptable. However, this does not preclude that, with an RbG study involving variants that cause disease recessively, re-contact can create tensions and anxiety in potential study participants.

### Stakeholder engagement

Studies that did not foresee RbG in their original consent should engage participants and other stakeholders in the design of an appropriate RbG policy. In our experience, stakeholder contribution was very valuable for the development of practical solutions addressing the ethical issues raised by RbG studies as well as for designing suitable research tools for investigating the attitudes and expectations of participants. We recommend consulting stakeholders in the design phase of such a study, as they can provide valuable insights for both the design of tailored strategies and general issues. We consulted several key partners in connecting the CHRIS scientists to the participants, including clinical geneticists, already involved in the CHRIS study when genetic counselling is needed, the ethics board, which is an essential part of the governance of the CHRIS study, and GPs, which play an important role in liaising with the community. CHRIS participants were engaged at different levels. Findings from a previously conducted empirical study on return of research results provided the basis for the investigation of participants’ views on RbG. Conducting an empirical study embedded in an RbG scientific study allowed us to obtain direct feedback from participants on their participation in an RbG study.

If feasible (in terms of resources and time constrains, number of recruited participants, and design of the study), such an embedded empirical study design would be a valuable strategy for shaping policy, because findings will be useful for understanding the specific context of the study, while designing and implementing an appropriate dynamic adjustment of the RbG policy. In addition, an embedded study design necessitates a close collaboration between researchers with different professional expertise (such as basic or clinical researchers and specialists in ethics), thus having also a bidirectional educational impact. By increasing awareness of the challenges posed by the re-contact of participants and the disclosure of individual genetic information, scientists be better able to reflect productively upon their own study design. The sharing of experiences, solutions, and tools from RbG studies should be widely encouraged. It is in this spirit we report in our pilot study and the steps we took in the process of a CHRIS RbG policy and offer the tools and insights gained therein.

## Conclusion

An RbG policy should balance the duty to provide information on the scientific rationale for recruitment with the right of the study participants to not to know sensitive information, while also trying to minimize the risk of deceiving participants by not informing them properly. Here, we outline the steps that we undertook in the policy building process for the CHRIS study. The difficulties in developing an acceptable and practicable RbG policy are to be found in those aspects of disclosing genetic information (informed consent, communication, and recruitment). By reporting on our experience in designing the workflow for the RbG pilot study, we aim to contribute to the design of best practices and tailored policy, and to participate in the reflection on research methodology in the field of RbG studies.

Our approach in understanding how best to design and conduct RbG studies in an ethically acceptable way and potentially draft policy and guidelines for best practices in RbG, included different steps and addressed the main ethical issues from different perspectives, which included reviewing the literature, consulting stakeholders, and collecting feedback from participants through an empirical study. The research on RbG best practices and policy we conducted and described here aimed to respond to different interests at stake: those of scientists, interested in conducting genetic studies to functionally understand the effects of genetic variants in health and disease, and those of participants, interested in having their rights respected and their wishes and expectations addressed, while participating in research. Based upon the insights gained through this policy-development process, we are currently working on a draft for a comprehensive RbG policy for future use and additional testing in CHRIS-based RbG studies.

## Supplementary information

Supplementary information 1

## References

[1] Corbin LJ, Tan VY, Hughes DA, Wade KH, Paul DS, Tansey KE (2018). Formalising recall by genotype as an efficient approach to detailed phenotyping and causal inference. Nat Commun..

[2] Beskow LM, Fullerton SM, Namey EE, Nelson DK, Davis AM, Wilfond BS (2012). Recommendations for ethical approaches to genotype-driven research recruitment. Hum Genet..

[3] Beskow LM, Linney KN, Radtke RA, Heinzen EL, Goldstein DB (2010). Ethical challenges in genotype-driven research recruitment. Genome Res..

[4] Garante per la protezione dei dati personali. General authorisation no. 8/2014 for the processing of genetic data. Garante per la protezione dei dati personali; 2014. https://www.garanteprivacy.it/web/guest/home/docweb/-/docweb-display/docweb/3831387. Accessed 14 Dec 2020.

[5] Doernberg S, Hull SC (2017). Harms of deception in FMR1 premutation genotype-driven recruitment. Am J Bioeth..

[6] Beskow LM (2017). Genotype-driven recruitment and the disclosure of individual research results. Am J Bioeth..

[7] Taylor HA, Morales C, Wilfond BS (2017). Genotype-driven recruitment in population-based biomedical research. Am J Bioeth..

[8] Amendola LM, Lautenbach D, Scollon S, Bernhardt B, Biswas S, East K (2015). Illustrative case studies in the return of exome and genome sequencing results. Pers Med..

[9] Papaz T, Liston E, Zahavich L, Stavropoulos DJ, Jobling RK, Kim RH (2019). Return of genetic and genomic research findings: experience of a pediatric biorepository. BMC Med Genom..

[10] Patch C, Middleton A (2018). Genetic counselling in the era of genomic medicine. Br Med Bull..

[11] Thorogood A, Dalpe G, Knoppers BM (2019). Return of individual genomic research results: are laws and policies keeping step?. Eur J Hum Genet..

[12] Robinson JO, Slashinski MJ, Wang T, Hilsenbeck SG, McGuire AL (2013). Participants’ recall and understanding of genomic research and large-scale data sharing. J Empir Res Hum Res Ethics..

[13] Beskow LM, Check DK, Namey EE, Dame LA, Lin L, Cooper A (2012). Institutional review boards’ use and understanding of certificates of confidentiality. PLoS ONE..

[14] Tan GD, Neville MJ, Liverani E, Humphreys SM, Currie JM, Dennis L (2006). The in vivo effects of the Pro12Ala PPARgamma2 polymorphism on adipose tissue NEFA metabolism: the first use of the Oxford Biobank. Diabetologia..

[15] Minion JT, Butcher F, Timpson N, Murtagh MJ (2018). The ethics conundrum in Recall by Genotype (RbG) research: perspectives from birth cohort participants. PLoS ONE..

[16] Budin-Ljosne I, Soye KJ, Tasse AM, Knoppers BM, Harris JR (2013). Genotype-driven recruitment: a strategy whose time has come?. BMC Med Genom..

[17] Pattaro C, Gogele M, Mascalzoni D, Melotti R, Schwienbacher C, De Grandi A (2015). The Cooperative Health Research in South Tyrol (CHRIS) study: rationale, objectives, and preliminary results. J Transl Med..

[18] Kaye J, Curren L, Anderson N, Edwards K, Fullerton SM, Kanellopoulou N (2012). From patients to partners: participant-centric initiatives in biomedical research. Nat Rev Genet..

[19] Michie M, Cadigan RJ, Henderson G, Beskow LM (2012). Am I a control?: genotype-driven research recruitment and self-understandings of study participants. Genet Med.

[20] Tabor HK, Brazg T, Crouch J, Namey EE, Fullerton SM, Beskow LM (2011). Parent perspectives on pediatric genetic research and implications for genotype-driven research recruitment. J Empir Res Hum Res Ethics..

[21] Beskow LM, Namey EE, Cadigan RJ, Brazg T, Crouch J, Henderson GE (2011). Research participants’ perspectives on genotype-driven research recruitment. J Empir Res Hum Res Ethics..

[22] Cadigan RJ, Michie M, Henderson G, Davis AM, Beskow LM (2011). The meaning of genetic research results: reflections from individuals with and without a known genetic disorder. J Empir Res Hum Res Ethics..

[23] Namey EE, Beskow LM (2011). Epilepsy patient-participants and genetic research results as “answers”. J Empir Res Hum Res Ethics..

[24] Beskow LM, Namey EE, Miller PR, Nelson DK, Cooper A (2012). IRB chairs’ perspectives on genotype-driven research recruitment. IRB..

[25] Richard AC, Peters JE, Savinykh N, Lee JC, Hawley ET, Meylan F (2018). Reduced monocyte and macrophage TNFSF15/TL1A expression is associated with susceptibility to inflammatory bowel disease. PLoS Genet..

[26] Stacey D, Chen L, Howson JM, Mason AM, Burgess S, MacDonald S, et al. Elucidating mechanisms of genetic cross-disease associations: an integrative approach implicates protein C as a causal pathway in arterial and venous diseases. 2020. 10.1101/2020.03.16.20036822.

[27] Lancaster TM, Dimitriadis SL, Tansey KE, Perry G, Ihssen N, Jones DK (2019). Structural and functional neuroimaging of polygenic risk for schizophrenia: a recall-by-genotype-based approach. Schizophr Bull..

[28] Corbin LJ, Hughes DA, Chetwynd AJ, Taylor AE, Southam AD, Jankevics A (2020). Metabolic characterisation of disturbances in the APOC3/triglyceride-rich lipoprotein pathway through sample-based recall by genotype. Metabolomics..

[29] Lee BP, Lloyd-Laney HO, Locke JM, McCulloch LJ, Knight B, Yaghootkar H (2016). Functional characterisation of ADIPOQ variants using individuals recruited by genotype. Mol Cell Endocrinol..

[30] Alver M, Palover M, Saar A, Lall K, Zekavat SM, Tonisson N (2019). Recall by genotype and cascade screening for familial hypercholesterolemia in a population-based biobank from Estonia. Genet Med..

[31] Tuomi T, Nagorny CLF, Singh P, Bennet H, Yu Q, Alenkvist I (2016). Increased melatonin signaling is a risk factor for type 2 diabetes. Cell Metab..

[32] Geiger MJ, Domschke K, Homola GA, Schulz SM, Nowak J, Akhrif A (2016). ADORA2A genotype modulates interoceptive and exteroceptive processing in a fronto-insular network. Eur Neuropsychopharmacol..

[33] Oliva I, Guardiola M, Vallve JC, Ibarretxe D, Plana N, Masana L (2016). APOA5 genetic and epigenetic variability jointly regulate circulating triacylglycerol levels. Clin Sci..

[34] Wade KH, Chiesa ST, Hughes AD, Chaturvedi N, Charakida M, Rapala A (2018). Assessing the causal role of body mass index on cardiovascular health in young adults: Mendelian randomization and recall-by-genotype analyses. Circulation..

[35] Ware JJ, Timpson N, Davey Smith G, Munafo MR (2014). A recall-by-genotype study of CHRNA5-A3-B4 genotype, cotinine and smoking topography: study protocol. BMC Med Genet..

[36] Hellmich C, Durant C, Jones MW, Timpson NJ, Bartsch U, Corbin LJ (2015). Genetics, sleep and memory: a recall-by-genotype study of ZNF804A variants and sleep neurophysiology. BMC Med Genet..

[37] Schwartzentruber J, Foskolou S, Kilpinen H, Rodrigues J, Alasoo K, Knights AJ (2018). Molecular and functional variation in iPSC-derived sensory neurons. Nat Genet..

[38] CHRIS Team. Studio sulla salute in Alto Adige. Informazioni sullo studio. CHRIS Team; 2019. https://it.chris.eurac.edu/wp-content/uploads/sites/11/2020/04/03-25-CHRIS-brochure-IT_2020-002-1.pdf. Accessed 2 Oct 2020.

[39] Lek M, Karczewski KJ, Minikel EV, Samocha KE, Banks E, Fennell T (2016). Analysis of protein-coding genetic variation in 60,706 humans. Nature..

[40] Anheim M, Elbaz A, Lesage S, Durr A, Condroyer C, Viallet F (2012). Penetrance of Parkinson disease in glucocerebrosidase gene mutation carriers. Neurology..

[41] Klein C, Lohmann-Hedrich K, Rogaeva E, Schlossmacher MG, Lang AE (2007). Deciphering the role of heterozygous mutations in genes associated with parkinsonism. Lancet Neurol..

[42] Lubbe SJ, Bustos B, Hu J, Krainc D, Joseph T, Hehir J, et al. Assessing the relationship between monoallelic *PARK2* mutations and Parkinson’s risk. 2020. 10.1101/2020.06.26.20138172. 10.1093/hmg/ddaa273 PMC8033143 33448283

[43] Bruggemann N, Mitterer M, Lanthaler AJ, Djarmati A, Hagenah J, Wiegers K (2009). Frequency of heterozygous Parkin mutations in healthy subjects: need for careful prospective follow-up examination of mutation carriers. Parkinsonism Relat Disord..

