# Supplementary material for: Balancing scientific interests and the rights of participants in designing a recall by genotype study
Source: Eur J Hum Genet. 2021 May 13;29(7):1146–57. doi: 10.1038/s41431-021-00860-7 (PMC8298596; doi:10.1038/s41431-021-00860-7)
Supplement: Supplementary file 1 — Supplementary information 1 [file 41431_2021_860_MOESM1_ESM.pdf]

CHRIS
eurac research

AUTONOME PROVINZ BOZEN – SÜDTIROL | PROVINCIA AUTONOMA DI BOLZANO – ALTO ADIGE
PROVINZIA AUTONOMA DE BULSAN – SUDTIROL

Südtiroler Sanitätsbetrieb | Azienda Sanitaria dell'Alto Adige
Azienda Sanitera de Sudtirol

VORNAME NACHNAME
ADRESSE

Bozen/Schlanders, Juli 2018

Einladung zur vertiefenden neurologischen Untersuchung PAREGEN

Sehr geehrte/r VORNAME NACHNAME,

wir kontaktieren Sie als Teilnehmer an der **ersten Phase** der Südtiroler Gesundheitsstudie CHRIS mit Ihrer ersten Untersuchung im CHRIS-Zentrum vom TT.MM.JJJJ, bei der Sie uns mitgeteilt haben, Interesse an der Teilnahme bei Folgestudien zu haben.

Wir möchten Sie einladen an einer **vertiefenden Untersuchung** teilzunehmen, die sich eingehend mit neurologischen Fragestellungen beschäftigen wird. Die Folgestudie hat das Ziel, Mechanismen zu erforschen, die vor neurologischen Krankheiten schützen können, mit einem besonderen Fokus auf Varianten im Parkin-Gen. Nähere Hinweise zur Studie entnehmen Sie bitte aus den beiliegenden „Informationen für Studienteilnehmer PAREGEN".

Wir laden zu dieser vertiefenden Untersuchung eine kleinere Gruppe von CHRIS-Teilnehmern ein, die **Unterschiede im Parkin-Gen** aufweisen: ein Teil der Probanden hat an einer Stelle im Parkin-Gen die übliche Standardsequenz, während ein anderer Teil auf einem der beiden Chromosomen eine Variante aufweist. Die Gruppenzugehörigkeit wird Ihnen nicht mitgeteilt, da keine unmittelbaren individuellen Konsequenzen bekannt sind.

Die nicht-invasiven Untersuchungen dauern insgesamt zwei Stunden. Die Teilnahme beinhaltet eine **erweiterte neurologische Untersuchung**, eine **Ultraschalluntersuchung** des Gehirns, eine einfache **Blutprobe** und eine detaillierte **Befragung**. Alle Untersuchungen werden von qualifiziertem und spezifisch geschultem Personal im Krankenhaus Schlanders durchgeführt. Sofern für Ihre Gesundheit relevante Ergebnisse auftreten sollten, werden die Befunde der klinischen Untersuchung Ihnen mitgeteilt und in Kopie übergeben.

In den kommenden Tagen wird Sie das CHRIS-Team telefonisch kontaktieren, um Ihnen einen Termin vom 20. bis 24. August 2018 vorzuschlagen. Ihre Teilnahme ist wie immer völlig freiwillig und kostenlos. Alle Ihre Daten werden sicher und streng vertraulich behandelt.

Wenn Sie es vorziehen, uns selbst zu kontaktieren, bitte wir um Ihre Rückmeldung unter

Tel. **0471 055 502** (Mo.-Fr.)
E-Mail **chris@eurac.edu**
Online **de.chris.eurac.edu/paregen**

Bitte kontaktieren Sie uns auch bei jeglichen Bedenken oder Rückfragen, die unsere Studie betreffen.

Wir sind überzeugt das Ihre Teilnahme ein wichtiger Beitrag zur neurologischen Gesundheitsforschung ist und hoffen, Sie bald im Krankenhaus Schlanders begrüßen zu dürfen.

Mit freundlichen Grüßen

Eurac Research, Institut für Biomedizin

Prof. Dr. habil. **Peter Pramstaller**, *Institutsleiter*

Dr. **Helmuth Weiss** (Innere Medizin, Krankenhaus Schlanders), *CHRIS-Studienarzt*

Univ.-Prof. Dr. **Christine Klein** (Universität Lübeck), *Klinische Leiterin der Folgestudie*

Das **CHRIS-Team** in Schlanders und Bozen

Vertiefende neurologische Untersuchung

Information für Studienteilnehmer PAREGEN

1. EINFÜHRUNG

Worum handelt es sich in vorliegendem Forschungsprojekt?

In den letzten Jahren hat sich gezeigt, dass sich im Rahmen von neurogenetischen Erkrankungen der Zusammenhang zwischen Genotyp und Phänotyp komplexer darstellt als zuvor vermutet: es konnten zahlreiche Träger von vermeintlich krankheitsverursachenden Mutationen identifiziert werden, die keine Zeichen der Erkrankung aufweisen. Viele Menschen tragen solche Varianten. Entweder sind die Symptome bei diesen Menschen sehr schwach oder sie weisen bestimmte Faktoren auf, die sie vor möglichen Krankheiten schützen. Dieses Phänomen wird als reduzierte Penetranz bezeichnet. Das Ziel dieser Studie ist es, Mechanismen zu untersuchen, die vor Krankheiten schützen können, mit einem besonderen Fokus auf Varianten im Parkin-Gen. Varianten in diesem Gen können bei Auftreten von zwei Mutationen (homozygot) eine genetische Parkinson-Krankheit verursachen, und es gibt Hinweise darauf, dass das Tragen einer einzelnen Mutation (heterozygoter Zustand) das Risiko für das Auftreten einiger abgeschwächter klinischer Symptome erhöhen kann.

Wo wird die Studie durchgeführt und wer wird um eine Teilnahme gefragt?

Diese Studie ist eine Folgestudie der CHRIS-Studie, eine vom Institut für Biomedizin (Eurac Research) und den Südtiroler Gesundheitsbetrieben durchgeführte longitudinale Studie, zu der die erwachsene Bevölkerung aus dem Vinschgau in Südtirol systematisch eingeladen wird. Die Studie wird somit am CHRIS-Studienzentrum im Krankenhaus Schlanders durchgeführt.

CHRIS-Studienteilnehmer sind grundsätzlich gesunde Teilnehmer an einer Bevölkerungsstudie und weitere phänotypische Informationen werden benötigt, um die Informationen von genetischen Daten besser interpretieren zu können. Es werden anhand von Genotypen im Parkin-Gen ausgewählte Probanden eingeladen: genotypisch unauffällige Probanden und Probanden mit einer einzelnen heterozygoten Variante im Parkin-Gen. Die Gruppenzugehörigkeit wird nicht mitgeteilt, da damit keine unmittelbaren individuellen Konsequenzen verbunden sind. Unsere Forschungen zielen darauf ab, schützende Faktoren zu identifizieren, im Moment haben wir aber noch keine Ergebnisse, aus denen sich ein klinischer Nutzen für Sie ergeben könnte.

2. UNTERSUCHUNGSPROGRAMM

Das Untersuchungsprogramm umfasst folgende Teile:

- Klinisch-neurologische Untersuchung (MDS-UPDRS III und IV, inkl. Videoaufzeichnung)
- Durchführung einer transkraniellen Sonografie (Ultraschalluntersuchung des Gehirns)
- Erhebung eines PD-spezifischen Minimal-Datensatzes (MDS-UPDRS I und II)
- Schriftprobe und Spiralenzeichnen
- Ganganalyse mit dem Mobility Lab
- Geruchtstest
- Einfache Blutprobe (für die Anlegung von Zellmodellen)
- Befragung zu persönlichen Eindrücken und Empfinden bezüglich der Studie/Untersuchung

Die Untersuchungen werden von qualifiziertem und spezifisch geschultem Personal im Krankenhaus Schlanders durchgeführt.

Erhalte ich die Ergebnisse der Untersuchung?

Die Befunde der klinischen Untersuchung werden Ihnen gleich nach der Untersuchung mitgeteilt, sofern für Ihre Gesundheit relevante Ergebnisse auftreten sollten.

Welche Art von Forschung wird durchgeführt und welche Rolle spielt dabei meine DNA und meine genetischen Daten?

Die Analyse der Erbsubstanz der CHRIS-Studienteilnehmer, die im Rahmen der CHRIS Studie durchgeführt wurde, hat zur Identifizierung von Probanden mit einzelnen heterozygoten Genvarianten im Parkin-Gen geführt. In dieser Studie möchten wir besser verstehen, welche Bedeutung diese heterozygoten Varianten für die Gesundheit haben und Faktoren identifizieren, die vor Krankheiten schützen können. Dazu ist eine standardisierte neurologische Untersuchung, ein transkranieller Ultraschall sowie die Beurteilung durch einen erfahrenen Neurologen vorgesehen.

Erhalte ich die Ergebnisse dieser Analysen?

Diese Studie dient ausschließlich dazu, das medizinisch-genetische Wissen über die untersuchte Krankheit zu verbessern. Wir informieren Sie, dass Ihre Blutprobe infolge der genetischen Informationen nicht nur über *Ihre* Gesundheit, sondern auch über die Ihrer Familienmitglieder Aufschluss geben könnte. **Die Studie wird aber ausschließlich zu Forschungszwecken durchgeführt.** Die Ergebnisse werden Ihnen nicht mitgeteilt, da sie für eine persönliche genetische Beratung nicht geeignet sind. Die Ergebnisse werden in anonymisierter Form wissenschaftlich ausgewertet.

Gibt es Risiken?

In dieser Studie werden nur Untersuchungen durchgeführt, die mit keinerlei Risiko für Ihre Gesundheit verbunden sind. Nach der Blutentnahme kann es in seltenen Fällen zu einem kleinen Hämatom (Bluterguss) an der Entnahmestelle kommen.

Aufklärung und Freiwilligkeit

Die Studienteilnahme ist freiwillig. Der Teilnehmer wird sein schriftliches Einverständnis zur Teilnahme geben, nachdem er die Studieninformation ausführlich gelesen und verstanden hat. Die Einverständniserklärung kann jeder Zeit widerrufen werden. Der Widerruf geschieht durch eine schriftliche oder telefonische Mitteilung an den Verantwortlichen der Datenverarbeitung und bringt keinerlei Nachteile für den Teilnehmer mit sich.

3. AUFBEWAHRUNG VON BIOLOGISCHEM MATERIAL UND DURCHFÜHRUNG DER STUDIE

Wie bei der CHRIS Studie werden aus ihrer Blutprobe Zellen extrahiert und in Kultur gebracht. Diese Zelllinien (immortalisierte Blutzellen und induzierte pluripotente Stammzellen, die zu Neuronen differenziert werden können) werden mit Hilfe einer Zahlencodierung codifiziert und in der Biobank des Instituts für Biomedizin an der Eurac Research in Bozen für den Zeitraum von 30 Jahren aufbewahrt und für Forschungsvorhaben verwendet.

4. FINANZIERUNG DER STUDIE

Die Kosten dieser Studie trägt das Institut für Biomedizin der Eurac Research, finanziert von der Autonomen Provinz Bozen - Südtirol, Abteilung Bildungsförderung, Universität und wissenschaftliche Forschung. Darüber hinaus wird diese Studie von der Deutschen Forschungsgemeinschaft (DFG): Forschergruppe FOR 2488 (Prof. Dr. Christine Klein, Universität Lübeck, Deutschland) für das Projekt "Reduced penetrance in hereditary movement disorders: Elucidating mechanisms of endogenous disease protection" gefördert.

5. DATENSCHUTZ UND MÖGLICHKEIT ZU FOLGESTUDIEN

Wie ist die Einhaltung der Datenschutzbestimmungen und der Privacy gesichert?

Im Sinne des Gesetzesdekret n. 196/03 bezüglich des Schutzes von Personen bei der Verwendung persönlicher Daten sowie im Sinne der **VERORDNUNG (EU) 2016/679 DES EUROPÄISCHEN PARLAMENTS UND DES RATES** informieren wir Sie, dass Ihre persönlichen Daten entsprechend der Datenschutzbestimmungen gesammelt und aufbewahrt und ausschließlich zu Forschungszwecken verwendet werden. Sie haben bei Ihrer ersten Teilnahme an der CHRIS-Studie entschieden, wie Ihre Daten behandelt werden sollen. Ihre Zustimmung ist noch gültig und Sie können sie im persönlichen Bereich „MY CHRIS“ auf der Internetseite jederzeit widerrufen oder abändern. Wie in der CHRIS Studie, werden auch in dieser Folgestudie Ihre medizinischen Daten und das biologische Material mit Hilfe einer

Zahlencodierung pseudonymisiert und getrennt von den persönlichen Daten, wie z.B. Name und Adresse, aufbewahrt. Die Zuordnung ihrer persönlichen Daten zu den Forschungsergebnissen ist dann nur noch dem Studienleiter und den von ihm beauftragten Mitarbeitern möglich. Gesammelte Informationen werden als streng vertraulich betrachtet und unterliegen dem Berufsgeheimnis. Die Daten und das biologische Material werden eventuell nur in anonymisierten Form mit den Forschungspartnern ausgetauscht. Die Daten werden in anonymisierter Form in Fachzeitschriften veröffentlicht oder auf Tagungen vorgestellt. Falls Sie es wünschen, haben Sie das Recht zu erfahren, welche und wie die Informationen aufbewahrt werden.

6. ERGEBNISSE DER STUDIE

Für die Studienteilnahme ist keine Art von Vergütung vorgesehen. Langfristig ist zu erwarten, dass sich durch die Kenntnis genetischer Einflüsse auf die untersuchte Erkrankung Vorteile für die Gesundheit der Bevölkerung ergeben. Ein eventueller Gewinn aus dieser Studie wird ausschließlich für die Finanzierung weiterer biomedizinischer Forschungsprojekte in Südtirol verwendet werden.

7. RECHTE DES STUDIENTEILNEHMERS

Die Rechte des Teilnehmers bezüglich der ihn betreffenden Daten sind in der Einverständniserklärung zur CHRIS-Studie wie folgt beschrieben:

1. Die betroffene Person hat das Recht, Auskunft darüber zu erhalten, ob Daten vorhanden sind, die sie betreffen, auch dann, wenn diese noch nicht gespeichert sind; sie hat ferner das Recht, dass ihr diese Daten in verständlicher Form übermittelt werden.
2. Die betroffene Person hat das Recht auf Auskunft über
   a) Die Herkunft der personenbezogenen Daten;
   b) Den Zweck und die Modalitäten der Verarbeitung;
   c) Das angewandte System, falls die Daten elektronisch verarbeitet werden;
   d) Die wichtigsten Daten zur Identifizierung des Rechtsinhabers, der Verantwortlichen und des im Sinne von Artikel 5 Absatz 2 namhaft gemachten Vertreters;
   e) Die Personen oder Kategorien von Personen, denen die personenbezogenen Daten übermittelt werden können oder die als im Staatsgebiet namhaft gemachten Vertreter, als Verantwortliche oder als Beauftragte davon Kenntnis erlangen können.
3. Die betroffene Person hat das Recht,
   a) Die Aktualisierung, die Berichtigung oder, sofern interessiert, die Ergänzung der Daten zu verlangen;
   b) Zu verlangen, dass widerrechtlich verarbeitete Daten gelöscht, anonymisiert oder gesperrt werden; dies gilt auch für Daten, deren Aufbewahrung für die Zwecke, für die sie erhoben oder später verarbeitet wurden, nicht erforderlich ist;
   c) Eine Bestätigung darüber zu erhalten, dass die unter den Buchstaben a) und b) angegebenen Vorgänge, auch was ihren Inhalt betrifft, jenen mitgeteilt wurden, denen die Daten übermittelt oder bei denen sie verbreitet wurden, sofern sich dies

nicht als unmöglich erweist oder der Aufwand an Mitteln im Verhältnis zum geschützten Recht unvertretbar groß wäre.

8. RECHTSINHABER UND VERANTWORTLICHER DER DATENVERARBEITUNG

Eurac Research mit Sitz in Bozen, in Person des gesetzlichen Vertreters Prof. Roland Psenner, ist „Rechtsinhaber der Datenverarbeitung“. Um weitere Informationen zu erhalten, können Sie das CHRIS Studienzentrum kontaktieren. Das Ihnen vorgestellte Studienprotokoll wurde vom Ethikkomitee des Südtiroler Sanitätsbetriebs genehmigt.
